# Providing free heroin to addicts participating in research – ethical concerns and the question of voluntariness

**DOI:** 10.1192/pb.bp.113.046565

**Published:** 2015-02

**Authors:** Edmund Henden, Kristine Bærøe

**Affiliations:** 1 Oslo and Akershus University College of Applied Sciences, Norway; 2 University of Bergen, Norway

## Abstract

Providing heroin to people with heroin addiction taking part in medical trials assessing the effectiveness of the drug as a treatment alternative breaches ethical research standards, some ethicists maintain. Heroin addicts, they say, are unable to consent voluntarily to taking part in these trials. Other ethicists disagree. In our view, both sides of the debate have an inadequate understanding of ‘voluntariness’. In this article we therefore offer a fuller definition of the concept, one which allows for a more flexible, case-to-case approach in which some heroin addicts are considered capable of consenting voluntarily, others not. An advantage of this approach, it is argued, is that it provides a safety net to minimise the risk of inflicting harm on trial participants.

In the bioethics literature, there has been considerable debate as to whether giving heroin addicts legal access to free heroin in connection with research on the effectiveness of heroin prescription as a treatment alternative constitutes a breach of ethical research standards. The ethical issue here is that the researcher must obtain the informed consent of the study participants. For their consent to be valid, individuals must give it voluntarily. The question is whether consent can be said to have been given voluntarily if the person has heroin addiction (we are assuming, of course, that they are neither intoxicated nor experiencing withdrawal symptoms at the time of giving consent). Those who claim that it cannot argue that it is in the nature of heroin addiction for individuals to lose their ability to resist their desire for heroin. Since a loss of ability means heroin addicts cannot refuse offers of free heroin, neither can we presume that they can give voluntary consent to take part in research that involves giving them a choice of free heroin.^1^ According to those who maintain that consent given by heroin addicts can be valid, this argument is flawed. Several studies show that financial concerns, fear of arrest, values regarding parenthood and many other factors influencing decisions in general often persuade a person addicted to heroin to cease their drug-oriented behaviour.^2^ That heroin addicts frequently respond to such incentives means that they cannot have lost the ability to resist their desire for heroin. We can presume, therefore, that heroin addicts have the competence to give voluntary consent to take part in trials involving the drug.

We want to argue that both sides in this debate are mistaken. Although it is plausible that many – perhaps even most or all – heroin addicts have the ability to resist their desire to take heroin, the degree to which their consent is voluntarily given greatly depends on the wider social and psychological circumstances under which they choose whether to consent or not. Focusing on these circumstances rather than universal and hard-to-verify claims about abilities of resistance allows for a more flexible, case-to-case approach, one that does not rule out the possibility that while some heroin addicts might be competent to give voluntary consent, some might not. One advantage of this approach compared with the alternatives is that it provides a safety net to minimise the risk of inflicting harm on the individuals who participate in these kinds of studies.

Before presenting our argument, a note of caution is in order: we do not want to claim that the answer to the ethical question raised by research on heroin prescription as a treatment alternative is alone sufficient to determine whether or not such research should be carried out. Even if the issue of voluntary consent in heroin trials was problematic, it does not follow that prescribing heroin as a treatment alternative should necessarily be banned. If, for example, the risks to the participants were small or non-existent while the benefits outweighed such risks, strategies that circumvent the normal standards of consent should indeed be considered. One such strategy could be to appoint some surrogate authority who is not involved in the study (e.g. a family member) to ensure the best interests of the participant, or perhaps to relax competence-defining criteria.^1^ Whether prescribing heroin therapeutically is an effective way of treating heroin users needs to be determined empirically and will not be discussed here.

## Two concepts of ‘voluntariness’

What does it mean to say that a consent is voluntary? In the bioethics literature it is widely agreed that a person acts voluntarily if he or she wills the action (performs it intentionally) without being under the controlling influence of another person or condition.^3^ Controlling influences are divided into ‘external’ and ‘internal’ depending on whether they are caused by the intentional actions of other persons (such as different forms of coercion or manipulation) or lack of internal self-control, an incapacity typically associated with mental illness. Although this characterisation of voluntary action is both plausible and, no doubt, helpful in many situations in which clinicians have to assess a person’s capacity, we believe it is ill suited to explaining how certain social circumstances and the beliefs they inform might sometimes constrain choices. This is because the circumstances and beliefs might exert a controlling influence on the person, causing them to feel pressured into performing certain actions (like enrolling in clinical trials, for example), without necessarily removing their internal capacity for self-control. In fact, we believe research on the effectiveness of heroin prescription as a treatment alternative provides an illustration of this, as we try to show in this article. First, however, we need to introduce another concept of ‘voluntariness’ that comes from political and legal philosophy.^4^ This will allow us to explain how social circumstances and the beliefs they inform could deprive a person of their voluntariness without removing their internal capacity for self-control. We introduce it here with a view to suggesting an alternative – and, we believe, important – perspective on the effects addiction might be thought to have on the voluntariness of heroin addicts’ consent that has been largely ignored in the debate about this issue. It is worth noting that it relies on a consequentialist ethical theory, which some readers may have objections about. We cannot provide a full conceptual defence of this notion or its ethical foundations here. Consequentialism, however, is a widely used approach in much ethical analysis in the field of healthcare.

Very briefly, this alternative concept of voluntariness begins by distinguishing between three types of options in terms of ‘acceptability’, where the standard for the acceptability of options is an objective standard of well-being. First, there are options that one strongly dislikes, which one holds to be ‘unacceptable’ in the sense that they bring one’s well-being below a certain threshold. These are options that are thoroughly bad because they involve losses it would be unreasonable to expect anyone to bear. Second, there are options that are undesirable but not thoroughly bad, which one holds to be ‘acceptable’ in the sense that they bring one’s well-being above a certain threshold. These are options that have sufficient value to be choiceworthy. Finally, there are options that bring one’s well-being up to a high level and that one likes so much that one chooses them. Consider then the following plausible definition of voluntary choice: a choice is voluntary if it is not made because no other acceptable alternative options are available. This negative definition implies the existence of two types of situations in which a person makes a voluntary choice. First, there are situations in which she has at least two acceptable options and chooses one of them because, all things considered, she prefers one option to the other. Second, there are situations in which she has at least one option that she likes so much that she chooses it because of that, whether or not there are any acceptable alternative options. In neither of these cases is her reason for making her choice not having other acceptable alternative options. One implication of this concept of voluntariness is that whether a choice is voluntary or not depends not just on the person’s internal capacity for self-control, but crucially also on her *beliefs* about her options and hence actual motivation for making the choice. As we argue in the next section, we cannot rule out that the social circumstances typical of many chronic heroin addicts affect their beliefs about their options in a way that undermine the voluntariness of their consent even if they retain their capacity for self-control (for an extended version of this argument, see Henden, 2013).^5^

## Why the circumstances of heroin addicts might undermine the voluntary nature of their consent

To determine whether heroin addicts are able to give voluntary consent, assuming the understanding of voluntariness just outlined, we need to know something about their beliefs about their options. Of course, one difficulty is that heroin addicts are not all alike. Their individual circumstances including social and personal resources are likely to differ, and their beliefs about their options are therefore likely to differ as well. That being said, there is widespread consensus that heroin treatment is suited to a minority of heroin users as a second-line treatment for those individuals who do not respond to methadone or buprenorphine treatment delivered under optimal conditions.^6^ Thus, heroin trials have essentially sought to determine the therapeutic value of prescribing heroin to high-risk heroin users for whom such benefits cannot be expected or achieved by existing treatment options.^7^ When discussing the competence of heroin addicts to consent to participation in heroin trials we should therefore focus primarily on chronic addicts with a history of repeated treatment failure. The prevalence of health and social problems in this group of addicts is widely acknowledged.^7^ Major psychopathological studies of heroin users report rates of comorbidity that far exceed those of general population estimates. In addition to having high rates of comorbidity, it is well known that many individuals with chronic heroin addiction lead marginalised, impoverished lives, often associated with criminal activity, anxiety and high levels of risk. Can it be ruled out that such circumstances might create situations of constrained choice? We believe that it cannot. To see how such a situation could arise, consider first the option of obtaining heroin from the street. Many individuals reach a point in their chronic heroin addiction history in which their current lifestyles do not seem to them to be sustainable any longer; evidence of this is that many eventually seek help for their addiction. Presumably the costs of maintaining this lifestyle begin to exceed the benefits. Put in the terminology introduced in the last section, we might say that they come to consider a life centred on the procurement of heroin to be ‘unacceptable’ in the sense of no longer bringing their well-being above a certain threshold. Consider next the option of abstaining from heroin. For an action to be an acceptable option, it is not sufficient to have the ability or power to perform it. One must also *believe* one has that ability or power. There are many studies showing that mood disorders such as depression and anxiety lower belief in the person’s capabilities or perceived self-efficacy.^8^ Since there is a strong correlation between mood disorders and chronic heroin addiction, it is reasonable to assume that many heroin addicts harbour a low sense of self-efficacy and lack confidence in their ability to abstain from heroin.^9^ Chronic heroin addiction is associated with hopelessness about the future and a sense of powerlessness to influence the direction one’s life is going – reinforced by a history of failed attempts to abstain. Now, a lack of belief in one’s own ability is clearly detrimental to one’s will. Thus, according to a standard philosophical view, intentions involve plans of action and such plans, in order to be rational, require the belief that one has an acceptable chance of changing the world in ways one believes are for the better.^10^ Given this view, it would not be rational to form intentions one believes one is not going to carry out. The implication is that individuals with heroin addiction who have little belief in their capacity to abstain are likely to find it extremely difficult to form the intention to abstain. That is, since they believe they are going to fail if they try, they are likely to lack the will to abstain. Consequently, their commitment to changing their way of life may be low. Since believing one has reasons not to make an effort to exercise an ability (since one thinks it is futile) is not equivalent to lacking the ability, the problem here is not a lack of ability. The problem rather is an impairment of rational will due to a lack of belief in self-efficacy. It cannot be ruled out that such impairments of the will may lead some chronic heroin addicts to falsely believe abstinence is a non-option.

If this is correct, everything depends on the option of taking part in research. Will a person with chronic heroin addiction consider this option to have a high value, not just as an acceptable way of avoiding the hassle on the street or to get free heroin from a legal source, but as a way of improving their well-being so much that they choose it *because* of that? In fact, there appears to be little reason to think so. Many heroin addicts actually refuse to take part in these studies. In heroin trials in Switzerland, only a third of participants decided to take part when given the choice.^11^ One reason, presumably, is that they find the costs of keeping regular appointments with healthcare professionals too high. Chronic heroin addicts who do consent must judge these costs as less important than the benefits associated with obtaining free heroin. Presumably, they consider the value of obtaining free heroin to be sufficient to make participation in research acceptable, even if they do not consider the combined value of obtaining free heroin and taking part in research to be very high.

To sum up, it cannot be ruled out that many chronic heroin addicts for whom the problems of procuring the drug on the street are unacceptable, but for whom abstinence is not an option, believe they have only one option, which is to consent to research involving the medical provision of heroin. Since there is evidence to suggest that they might choose this option not because they like it very much, but because they have no acceptable alternative options, their consent could be construed as non-voluntary. What constrains their choice is not their desire for heroin, but the wider social and psychological circumstances of their heroin addiction and the beliefs about the options these circumstances create.

Our reasoning here is, of course, hypothetical. We have no empirical evidence of the contents of the decision-making processes in individuals with heroin addiction (nor do we know whether any such evidence exists). However, if it is a plausible reconstruction of what these processes might look like given the situational constraints and our current state of knowledge, it suffices, we believe, for our current purpose, to provide a reason why we should not take the voluntariness of their consent for granted.

## Voluntary consent and risk-minimising ethical analysis

As we have argued, given a certain plausible conception of voluntariness, we cannot rule out that the beliefs held by individuals with heroin addiction about their options and hence their motivation for action might undermine the voluntariness of their consent even if they have the ability to resist their desire for heroin. One important reason for this is that the wider social circumstances typical of many such individuals may shape their beliefs about their capacity to make choices (such as making abstinence seem impossible) and these beliefs (which may be false) could then impair their will to abstain from heroin use. This suggests that paying special attention to these circumstances should form an integral part of the assessment of whether or not the person’s consent is voluntary. However, the argument may perhaps strike some as overly philosophical. On what basis can we decide between different conceptions of voluntariness? Let us end with some more general considerations in favour of the view we have presented.

According to the World Medical Association’s declaration on ethical principles for medical research involving human subjects (the Helsinki Declaration), the participation of human subjects in research requires the voluntary consent of individuals who are considered competent to give their consent (www.wma.net/en/30publications/10policies/b3/). What makes it so ethically challenging to assess whether, in the case of heroin research, a person is competent to give their consent voluntarily is, of course, that we have no uncontroversial concept of voluntariness that applies to the specific circumstances of heroin addicts, and we have no direct access to the mental processes of the consenting individual to accurately appraise whatever conception we lean towards. This means that judgements about voluntary consent will always involve uncertainty. How can we best deal with this uncertainty? An ethical way of justifying why one approach is chosen over another might be to compare the potential harm these approaches may inflict on the research participants. Such an overall account of harm will have to reflect the inherent uncertainty of the assessment and also include considerations of harm potentially caused by a flawed assessment.

In this article we have identified three different approaches to consent in individuals with heroin addiction:

- a person’s desire for heroin rules out any ability to choose freely between receiving heroin or not, hence we should presume that no heroin addict can voluntarily consent to medically prescribed heroin;
- heroin addicts have the ability to choose freely between receiving heroin or not, hence we should presume that all heroin addicts can voluntarily consent to medically prescribed heroin;
- the social and psychological circumstances of some individuals with heroin addiction might be such that we cannot presume that they can voluntarily consent to medically prescribed heroin.

Which of these approaches would minimise the harm inflicted on the person if they were used to inform an assessment of their competence to give voluntary consent?

Consider (a). There may be circumstances in which it might be better for some individuals with heroin addiction to receive free heroin under medical supervision than getting it on the street. That is, the harm inflicted on these addicts by obtaining heroin on the street might greatly exceed the potential harm resulting from participation in heroin trials, because of mistaken assumptions about valid consent. Thus, these addicts might end up worse off than if (a) had not been used as the basis for an assessment of voluntary consent. Ironically, the protective safety net of the ethical standard of valid consent breaks down in this case, and in fact inflicts more harm than if the standard were ignored. Next, consider (b). The circumstances of many individuals addicted to heroin might suggest that they have some chance of succeeding in abstinence-based drug treatment programmes. However, this option of trying to achieve a life free of heroin is effectively ruled out if they receive heroin medication on a regular basis. Consequently, the harm these individuals may suffer could be considerable if their consent is accepted as valid without further questioning. Again, they could end up worse off than if we had not used (b) as the basis of the assessment of voluntary consent. Finally, consider (c). This approach differs from (a) and (b) by focusing on the particular person’s social and psychological circumstances (including motivating beliefs) as the basis of the assessment, rather than on universal and hard-to-verify claims about abilities of resistance of persons with heroin addiction. It therefore allows for a more flexible, case-to-case approach, one that neither rules out competence to consent voluntarily nor rules it in. This option would minimise the risk of inflicting more harm than if (c) had not been applied. Consequently, the potential of inflicting harm by assuming this approach is smaller compared with (a) and (b).

## Conclusion

Philosophy and medicine are inherent to mental healthcare. Clinical assessments of mental non-observable categories rely on adequate philosophical conceptualisations. Since the adequacy of these conceptualisations cannot be settled *a priori* and uncertainty will always be involved whenever attempts are made to confirm or reject their appropriateness *a posteriori*, philosophy offers a means of identifying the most apt conceptualisation according to a risk-minimising ethical analysis. An assessment of capacity for voluntary consent in individuals with heroin addiction should be based on an approach that minimises the risk of harming them more than if the approach were not applied. According to our argument, focusing on addicts’ social and psychological circumstances (including motivating beliefs) as the basis of an assessment rather than their abilities of resistance is the most apt approach in this regard. We therefore suggest that this approach to the assessment of participant consent should guide and inform an ethical practice of including and excluding heroin addicts in research on heroin provision.
